# Nucleoprotein Nanostructures Combined with Adjuvants Adapted to the Neonatal Immune Context: A Candidate Mucosal RSV Vaccine

**DOI:** 10.1371/journal.pone.0037722

**Published:** 2012-05-24

**Authors:** Aude Remot, Xavier Roux, Catherine Dubuquoy, Jenna Fix, Stephan Bouet, Mohammed Moudjou, Jean-François Eléouët, Sabine Riffault, Agnès Petit-Camurdan

**Affiliations:** 1 Molecular Virology and Immunology (UR892), French National Institute for Agricultural Research, Jouy-en-Josas, France; 2 Animal Genetics and Integrative Biology (UMR1313), French National Institute for Agricultural Research, Jouy-en-Josas, France; University of Georgia, United States of America

## Abstract

**Background:**

The human respiratory syncytial virus (hRSV) is the leading cause of severe bronchiolitis in infants worldwide. The most severe RSV diseases occur between 2 and 6 months-of-age, so pediatric vaccination will have to be started within the first weeks after birth, when the immune system is prone to Th2 responses that may turn deleterious upon exposure to the virus. So far, the high risk to prime for immunopathological responses in infants has hampered the development of vaccine. In the present study we investigated the safety and efficacy of ring-nanostructures formed by the recombinant nucleoprotein N of hRSV (N^SRS^) as a mucosal vaccine candidate against RSV in BALB/c neonates, which are highly sensitive to immunopathological Th2 imprinting.

**Methodology and Principal Findings:**

A single intranasal administration of N^SRS^ with detoxified *E.coli* enterotoxin LT(R192G) to 5–7 day old neonates provided a significant reduction of the viral load after an RSV challenge at five weeks of age. However, neonatal vaccination also generated an enhanced lung infiltration by neutrophils and eosinophils following the RSV challenge. Analysis of antibody subclasses and cytokines produced after an RSV challenge or a boost administration of the vaccine suggested that neonatal vaccination induced a Th2 biased local immune memory. This Th2 bias and the eosinophilic reaction could be prevented by adding CpG to the vaccine formulation, which, however did not prevent pulmonary inflammation and neutrophil infiltration upon viral challenge.

**Conclusions/Significance:**

In conclusion, protective vaccination against RSV can be achieved in neonates but requires an appropriate combination of adjuvants to prevent harmful Th2 imprinting.

## Introduction

Human respiratory syncytial virus (hRSV) is a major cause of severe lower respiratory tract infections in infants less than 6 months and in immuno-compromised or elderly patients [1], [2], [3], [4]. Besides, infants who develop acute RSV bronchiolitis at an early age are at increased risk for prolonged wheezing and future development of asthma [5], [6] through excessive priming of Th2 cells [7]. Bovine RSV (bRSV) is a closely related pneumovirus, also causing severe and sometimes fatal respiratory disease in calves [8].

Veterinary RSV vaccines exist but could be improved, whereas several factors have impeded the development of an effective and safe hRSV vaccine [9], [10]. First of all, concerning a pediatric RSV vaccine, safety will be of peculiar concern since, in the 60's, a vaccination trial with formaldehyde-inactivated virus in alum (FI-RSV) led to an exacerbated disease upon seasonal RSV infection in most vaccine recipients and two infants died with a prominent pulmonary neutrophilia and moderate eosinophilia [11], [12]. Extensive studies using rodent models have attributed disease exacerbation to immunopathological responses: in adult BALB/c mice, Th2 biased immune responses to the FI-RSV vaccine lead, upon RSV challenge, to a pro-inflammatory “cytokine/chemokine storm” promoting excessive pulmonary leukocyte infiltration, with prominent eosinophilia, and goblet cell hyperplasia [13]. Second, apart from the elderly, one major target population for vaccination is the newborn or very young infant, with its relatively immature innate and adaptive immune system prone to Th2-biased immune responses [14], and potential interference of maternal antibodies [15]. Third, adjuvants and/or delivery modes best adapted to elicit protective and non pathogenic mucosal immunity in such very young infants have yet to be improved and licensed. Interestingly, studies in neonatal mice have suggested ways to reduce the Th2 bias of neonatal responses to sub-unit protein vaccines, like the use of CpG oligodeoxynucleotides as adjuvant [16], [17].

Most of the RSV vaccines presently under development are targeting the two surface glycoproteins F and G, which bear epitopes recognized by neutralizing antibodies [10]. Much less has been done to explore the potential interest as a vaccine component of the nucleoprotein (N), although it was recognized as one of the targets of T cell immunity, inducing both helper and cytotoxic T cells in human [18], [19]. Besides, N is highly conserved between hRSV subtypes and even bears >85% amino-acid homology with N from bRSV, so it could be an interesting component of a heterosubtypic vaccine. Indeed, the combination of plasmids encoding the RSV N and F proteins administered to calves or infant rhesus monkeys was shown to provide protection without causing disease exacerbation [20], [21].

An original process was developed in our laboratory, allowing to produce and purify large amounts of recombinant N from hRSV as soluble ring structures composed of 10–11 N monomers bound to random stretches of bacterial RNA (70 bp), which we named N^SRS^ (for Sub-nucleocapsid Ring Structure) [22], [23]. In a recent paper, we documented its immunogenicity and vaccine potential, when administered to adult BALB/c mice with, as adjuvant, the mutant *E. coli* heat-labile toxin LT(R192G) (hereafter abbreviated as LT) [24]. Nasal vaccination with N^SRS^ and LT elicited strong local and systemic immunity characterized by high titers of anti-N antibodies (IgA in the broncho-alveolar lavage (BAL) and serum IgG1 and IgG2a), antigen specific CD8+ memory T cells and IFNγ producing CD4+ T cells in the absence of pathological lung inflammation upon RSV challenge [24]. The same antigen administered parenterally with a water-in-oil adjuvant (Seppic) to colostrum-deprived one-month-old calves proved partially protective against bRSV challenge, without inducing lung eosinophilia or adverse lung inflammation [25].

In the present study, we chose to test our new mucosal vaccine candidate in newborn (5 to 7 days old) BALB/c mice, a very relevant animal model to address the risk of immuno-pathological responses against RSV [26]. Indeed, a primary RSV infection in BALB/c neonates leads to airway disease exacerbation with lung eosinophilia upon re-infection at adult age [27]. Besides, FI-RSV vaccination of BALB/c neonates has a more severe immunopathological outcome upon virus challenge than the one elicited upon FI-RSV vaccination of adult BALB/c mice [28]. We confirmed the capacity of our vaccine to trigger efficient anti-viral immunity after just one nasal administration to 5–7 days old pups. However viral protection was associated with some disease exacerbation and airway infiltration with neutrophils and eosinophils upon RSV challenge, that appeared related to a Th2 neonatal imprinting. Addition of a second pro-Th1 adjuvant (CpG oligodeoxynucleotides) was able to restore a mixed Th1/Th2 immune memory and abrogated eosinophilia with only a minor reduction of viral protection upon challenge. Surprisingly, neonates treated with this combination of adjuvants without antigen turned out to be equally protected against virus replication.

## Results

### Neonatal nasal vaccination with N^SRS^ and LT(R192G) provided viral protection but exacerbated lung disease upon challenge

Five to seven days old pups received one intranasal (i.n.) administration of N^SRS^ (3 µg) with or without adjuvant LT (2 µg) (this vaccine formulation is noted thereafter N+LT). Mice vaccinated as neonates where challenged 4 to 5 weeks later with hRSV-A2. Mice were killed at day 5 post-challenge, which allowed us to monitor the viral load, the weight loss and the onset of the antibody response or lung inflammation in the same individual mice. Pups for one experimental group were chosen from at least two different litters and to reach a sufficient number of pups in each experimental group allowing statistical analysis, we combined data from several experiments (each experiment is shown with a different symbol).

**Figure 1:**
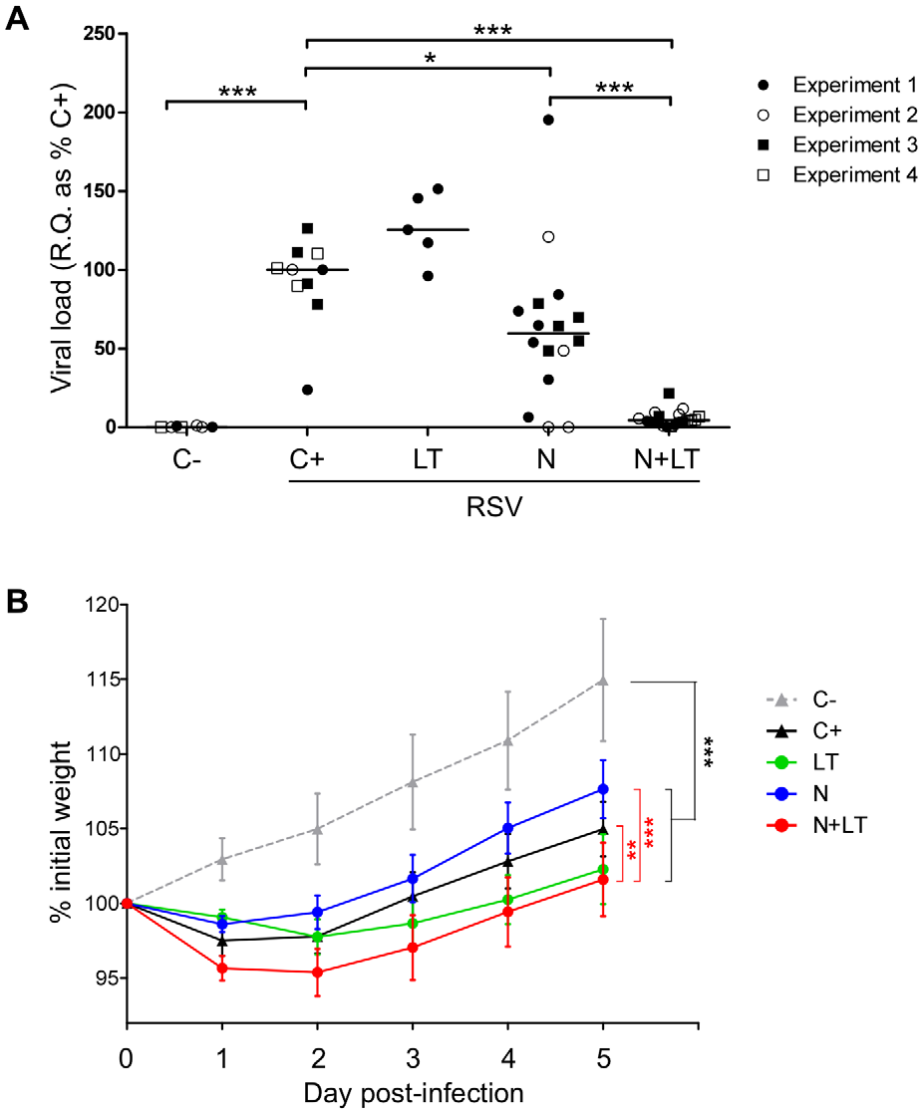
Neonatal nasal vaccination with N+LT conferred viral protection but exacerbated airway disease upon RSV challenge. Male and female pups (5–7 day-old) were vaccinated by intranasal instillation of 10 µL saline containing or not 3 µg N and 2 µg LT as indicated. At 5 weeks of age, mice were challenged by intranasal instillation of 50 µL (5.10^6^ pfu) hRSV A2. Controls included unvaccinated infected (C+) and uninfected (C−) littermates. Animals were killed 5 days post challenge (d5 p.i.). (A) Individual viral load assessed by qRT-PCR: R.Q. of N transcripts, normalized to HPRT, are expressed as % of the unvaccinated infected control group (C+) (R.Q. = 100×2^−ΔΔCt^). Four independent experiments combining different treatment groups are shown with ≥5 mice per group (C−: n = 7, C+: n = 10, LT: n = 5, N: n = 16, N+LT: n = 20). Mann-Whitney U-test was used for comparison between treatments (* p<0.05; *** p<0.001 and **** p<0.0001). (B) Mice were weighed daily from d0 till d5 p.i. and individual weight loss/gain was calculated as % of initial weight. Data are mean±SEM from n≥5 mice per group. Statistical analysis was performed to compare growth over the period d2 to 5 p.i. using the Tukey's multiple comparison test, repeated measures one way ANOVA (** p<0.01 and *** p<0.001).

A single neonatal N+LT i.n. immunisation led to a strong reduction of the viral mRNA load in the lungs 5 days after challenge (Fig. 1A, N+LT *versus* unvaccinated RSV-infected controls C+ p<0.001). Administered alone, LT did not reduce the viral load and N gave partial and highly variable protection (Fig. 1A, N *versus* C+ p<0.05).

As illustrated Fig. 1B, the RSV challenge induced a moderate but significant weight loss from day 2 up to day 5 post-infection in 5 weeks-old mice (all infected groups *versus* uninfected littermates [C−] p<0.001). Weight loss was more severe in the N+LT vaccinated group than in unvaccinated (C+) or N vaccinated mice (N+LT *versus* C+ p<0.01; N+LT *versus* N p<0.001). Neonatal vaccination with LT alone also led to increased weight loss in most mice (LT *versus* N p<0.001, but LT *versus* C+ or N+LT p>0.05) suggesting a contribution of LT to disease exacerbation.

**Figure 2:**
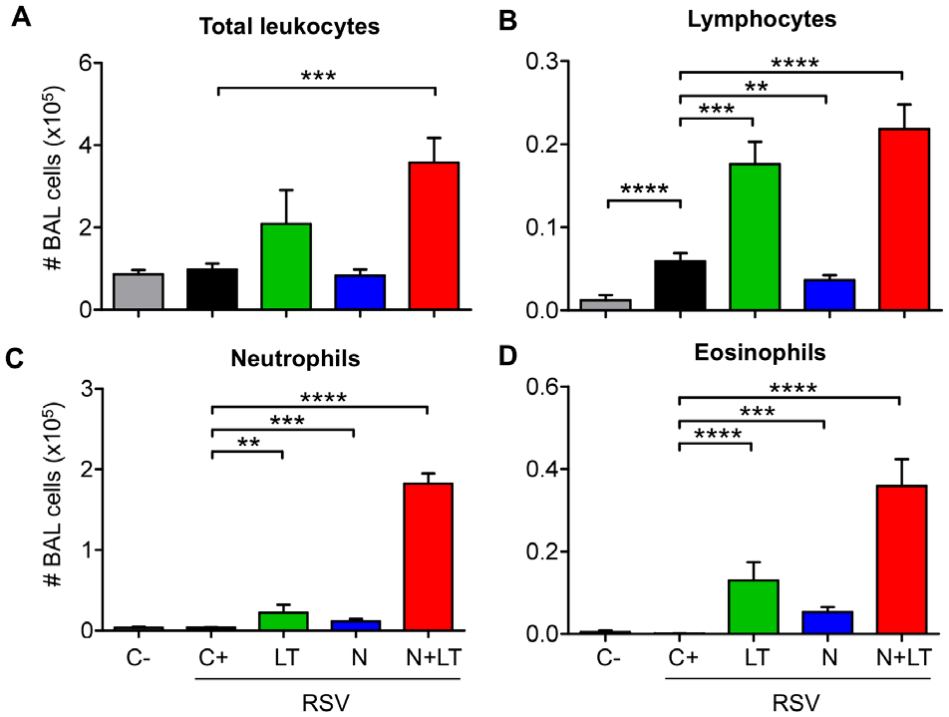
LT and N contributed to the inflammatory responses recorded in BAL after the RSV challenge. Mice vaccinated as neonates as in Fig.1 were killed 5 days post challenge (d5 p.i.). BAL cells were numerated, cytocentrifuged and stained with May-Gründwald-Giemsa (MGG). Data are mean±SEM from n≥10 mice per group for total leukocytes (A), lymphocytes (B), neutrophils (C) and eosinophils (D) numbers. Mann-Whitney U-test was used for comparison between treatments (** p<0.01; *** p<0.001 and **** p<0.0001).

Moreover, mice vaccinated as neonates with N+LT and challenged with RSV as young adults had significantly more leukocytes in their BAL at day 5 p.i. than unvaccinated infected controls (C+) or mice vaccinated with N alone (Fig. 2A: N+LT *versus* C+ *p*<0.001). Vaccination with LT alone also led to an increased number of BAL leukocytes in most mice upon challenge, although to a more variable and thus non significant level (LT *versus* C+ p>0.05).

Determination of BAL cell subtypes by May-Grünwald-Giemsa staining showed that the viral challenge *per se* did not alter significantly BAL cell composition, except for a minor but significant increase in the number of lymphocytes (Fig. 2B: C+ *versus* C− p<0.0001). Vaccination with N or LT alone led to a further and highly significant increase in BAL lymphocytes (LT *versus* C+ p<0.001; N *versus* C+ p<0.01), neutrophils (Fig. 2C: LT *versus* C+ p<0.01; N *versus* C+ p<0.001) and eosinophils (Fig. 2D: LT *versus* C+ p<0.0001, N *versus* C+ p<0.001). Combining N+LT in the neonatal vaccine further increased the number of lymphocytes, neutrophils and eosinophils (N+LT *versus* C+: p<0.0001 for the 3 subsets). Taken together, these data suggested that both LT and N contributed to the inflammatory responses recorded after the RSV challenge. As pulmonary eosinophilia was suggestive of a Th2 biased memory response to N, we explored the type of antiviral immunity primed by N+LT and investigated further whether it could be modulated.

### Neonatal vaccination with N+LT imprinted a Th2 biased N-specific immunity, which could be modulated with CpG

**Figure 3:**
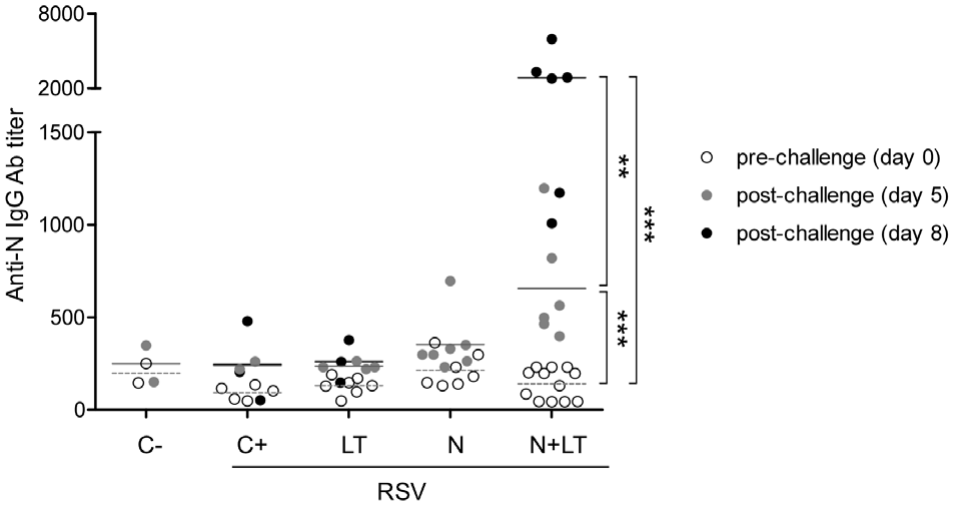
Neonatal N+LT nasal immunization primed for an early anti-N Ab response after the RSV challenge. Mice vaccinated as neonates as in Fig.1 were bled before (day 0) and 5 or 8 days after the viral challenge with hRSV-A2 (d5 or d8 p.i.). Serum anti-N antibody titers were assessed by an endpoint dilution ELISA assay on plates coated with N using HRPO-conjugated rabbit anti-mouse Ig(H+L) Abs. Individual titers and the mean titer for day 0 (white circles, dotted line), d5 p.i. (grey circles, plain line) and d8 p.i. (black circles, plain line) are figured. Man-Whitney U-test was used for comparison of titers at day 0, 5 and 8 p.i. for each group (** p<0.01; *** p<0.001).

Sera collected before (d0) and 5 or 8 days after the viral challenge (d5, d8 p.i.) were titrated by ELISA for anti-N antibodies. As illustrated Fig. 3, anti-N antibody titers were at baseline level in all groups before challenge (a month after neonatal vaccination) but increased as early as 5 days and even more so at 8 days after the RSV challenge in all N+LT vaccinated mice, suggesting a memory response.

To further explore this hypothesis, we amplified the primary neonatal response by a vaccine boost with N at 5 weeks. As shown in Table 1 for N+LT primed mice, serum antibodies detected 7 days after the boost were mostly of the IgG1 subclass and no anti-N IgA were detected in the BALF. To reduce the Th2 neonatal imprinting, we decided to implement our neonatal vaccine formulation with CpG (ODN-1826), a known pro-Th1 adjuvant in neonates [16]. Indeed, contrarily to their N+LT primed littermates, a few mice primed with N+CpG and most mice primed with N+LT+CpG had high titers of IgG2a anti-N serum antibodies, as well as detectable anti-N IgA in their BALF 7 days after the N boost (Table 1).

**Table 1:** Anti-N antibody responses primed by neonatal nasal vaccination shifted from IgG1 to IgG2a and IgA isotypes when CpG were added as adjuvant.

	Serum				BALF	
Prime(a)	IgG1		IgG2a		IgA	
N+LT	1694±836(b)	4/4(c)	53±19	1/4	≤3	0/5
N+CpG	493±395	3/6	3751±3397	1/6	5±2	1/6
N+LT+CpG	764±396	9/9	1523±1252	5/9	10±1	6/9
LT+CpG	46±12	2/8	≤30	0/8	≤3	0/2

(a) Neonates were immunised *i.n.* at age 5–7 days as indicated (prime) and received one intranasal boost with N (10 µg) at 5 weeks. Sera and BALF were collected 7 days after the boost.

(b) Antibody titers against N were determined by ELISA using endpoint dilution assay. Data are mean±SEM (≤30 or ≤3: not detected at the first tested dilution for sera or BALF).

(c) Number of responders/number tested.

Spleen and local lymph node (LN) cells, collected 7 days after a boost with N, were cultured for 72 h *in vitro* with N, or with PMA-Ionomycin and medium alone as positive and negative controls respectively. Culture supernatants were assayed by ELISA for IFNγ, a Th1 cytokine and IL-5, a Th2 cytokine. As illustrated Table 2, spleen and LN cells from N+LT primed pups boosted with N produced both IFNγ and IL-5, with the highest IL-5 titers being produced by LN cells in some mice, confirming that neonatal N+LT vaccination induced a Th2 biased local T cell memory. By contrast, spleen and LN cells from mice primed with N+LT+CpG or N+CpG produced higher titers of IFNγ than of IL-5, confirming that CpG added to the neonatal N+LT vaccine reoriented memory T cells towards Th1 (Table 2).

**Table 2:** Memory T cell responses primed by neonatal nasal vaccination shifted from Th2 to Th1 cytokine profile when CpG were added as adjuvant.

	Spleen(b)		LN(b)	
Prime(a)	IFNγ(c)	IL-5	IFNγ	IL-5
N+LT	654±159(d)	9±6	1431±650	511±361
N+CpG	614±20	<7±0	667±97	112±78
N+LT+CpG	2268±734	7±2	580±112	64±46
LT+CpG	357±98	7±3	182±123	8±5

(a) Neonates were immunised *i.n.* at age 5–7 days as indicated (prime) and received one intranasal boost with N (10 µg) at 5 weeks.

(b) Spleen and cervical lymph node (LN) were collected 7 days after the boost (LN were pooled from 2 to 3 mice). Single cell suspensions were cultured with N, PMA-ionomycin or medium for 72 hours.

(c) Culture supernatants were assayed by ELISA for IFNγ and IL5.

(d) Cytokine concentrations in supernatants from cultures restimulated with N (mean pg/mL ±SEM for n≥2 for spleen and pool of 2 for LN). (All cell cultures responded to PMA, producing 39838±8542 pg/mL IFNγ).

### Combining CpG and LT as adjuvants for neonatal vaccination provided protection against viral challenge without lung eosinophilia

In order to test whether CpG added to the neonatal N or N+LT vaccine could also prevent disease exacerbation after challenge, neonates vaccinated i.n. with N and/or LT and/or CpG were challenged with RSV a month later.

**Figure 4:**
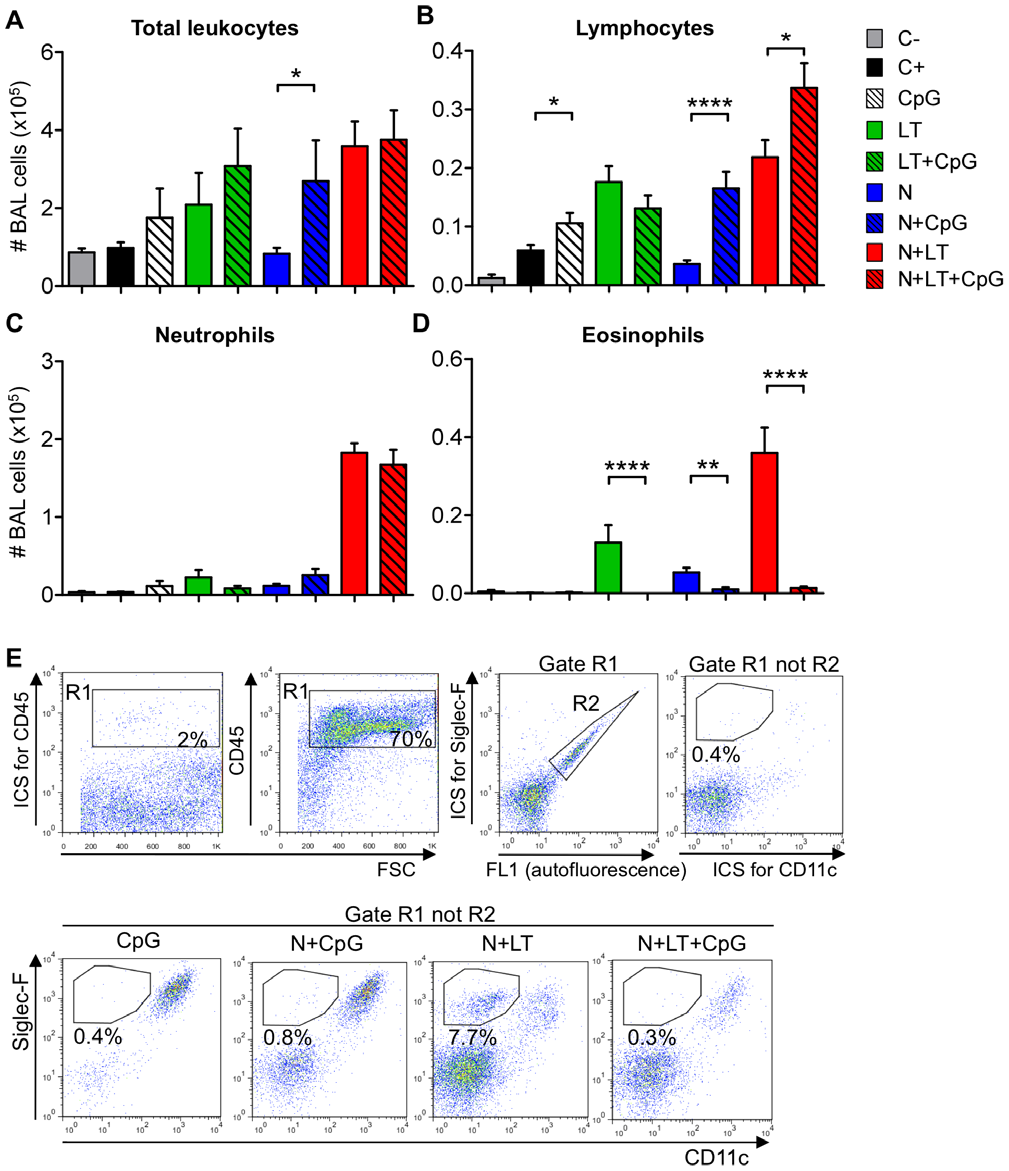
CpG added to the neonatal vaccine abrogated BAL eosinophilia upon RSV challenge. Mice vaccinated as neonates as in Fig.1, with or without addition of 2 nmoles CpG (ODN 1826), as indicated, were sacrificed 5 days after the viral challenge with hRSV-A2. BAL leukocytes were numerated, cytocentrifuged and stained with May-Grünwald-Giemsa (MGG). Data are mean±SEM from n≥10 mice per group combining three independant experiments. Mann-Whitney test was performed to compare the total number of leukocytes (A), as well as the number of lymphocytes (B), neutrophils (C) and eosinophils (D) (** p<0.01; *** p<0.001 and **** p<0.0001). (E) BAL cells from each experimental group were pooled (n≥5 mice per group) and stained for FACS analysis using Siglec-F-PE, CD45-PerCP and CD11c-Biotin monoclonal antibodies followed by Streptavidine APC. Isotype control stainings (ICS) were done with irrelevant isotype-matched antibodies on a pool of BAL cells from all experimental groups. After gating on CD45^+^ leukocytes (R1) and excluding autofluorescent cells (R2), eosinophils were detected as Siglec-F^+^ and CD11c^low^. Data analysis was performed using FlowJo software with at least 5.000 events in the (R1 not R2) gate. Dot plots represent one of two experiments with similar data.

Addition of CpG to each vaccine formulation tested (LT, N and N+LT) was associated with a moderate increase in the number of BAL leukocytes, that was not significant except between the groups vaccinated with N+CpG *versus* N (p<0.05, Fig. 4A). Addition of CpG led to a slight increase in the number of lymphocytes (except when added to LT alone, Fig. 4B), and, whereas it did not reduce the number of neutrophils (Fig. 4C), it nearly abolished BAL eosinophilia after challenge in each formulation (Fig. 4D, N+CpG *versus* N p<0.01, LT+CpG *versus* LT and N+LT+CpG *versus* N+LT p<0.0001). FACS analysis of BAL cell suspensions (Fig. 4E) confirmed that addition of CpG to the N+LT neonatal vaccine abolished BAL eosinophilia (from nearly 8% down to 0.3% SiglecF^+^ CD11c^low^ CD45^+^ BAL cells). As expected, BAL leukocytes were mostly alveolar macrophages (SiglecF^+^, CD11c^+^) for CpG vaccinated controls, whereas a variable proportion of BAL leukocytes were double negative (lymphocytes or neutrophils) for the N+CpG, N+LT or N+LT+CpG vaccinated groups.

**Figure 5:**
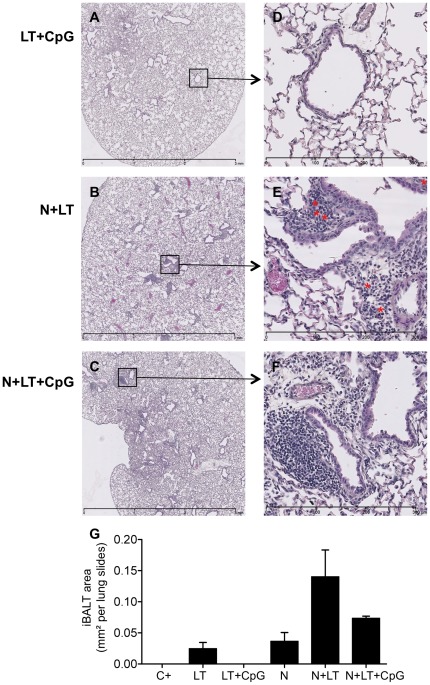
Neonatal nasal vaccination with N+LT or N+LT+CpG augmented cellular infiltration in lung tissue upon RSV challenge. Mice vaccinated as neonates as indicated or non vaccinated littermates were sacrificed 8 days after the hRSV-A2 challenge. Lung were dissected out, fixed, embedded in paraffin and sectioned at 5 µm. Lung sections were then stained with hematoxylin-eosin-saffron and photographed using a Nanozoomer (Hamamatsu). One representative section per group is shown (A–C: original magnification 2.5×) with red asterix figuring eosinophils in the enlarged selected area (D–F: enlargement 20×). (G) Total iBALT area was measured using the NDPview software (Hamamatsu) for one representative lung section of each mouse (n≥2 mice per group). Data are mean±SEM.

Histological examination of lungs collected 8 days post-challenge (Fig. 5) showed few diffuse peribronchiolar infiltrates in the lungs from C+ (data not shown) or LT+CpG vaccinated mice (Fig. 5A, 5D). On the opposite, lung sections from mice vaccinated with N+LT (Fig. 5B, 5E) or N+LT+CpG (Fig. 5C, 5F) displayed large peribronchiolar and perivascular immune infiltrates mostly composed of mononuclear cells, which clearly suggested the development of bronchus associated lymphoid tissue (iBALT). Although the average iBALT area/lung section (n≥2 mice/group) was not statistically different (Fig. 5G), eosinophils (indicated with red asterix) were frequently observed at the periphery of these infiltrates in lung sections from N+LT (Fig. 5E) but were rarely found in lung sections from N+LT+CpG vaccinated pups (Fig. 5F).

**Figure 6:**
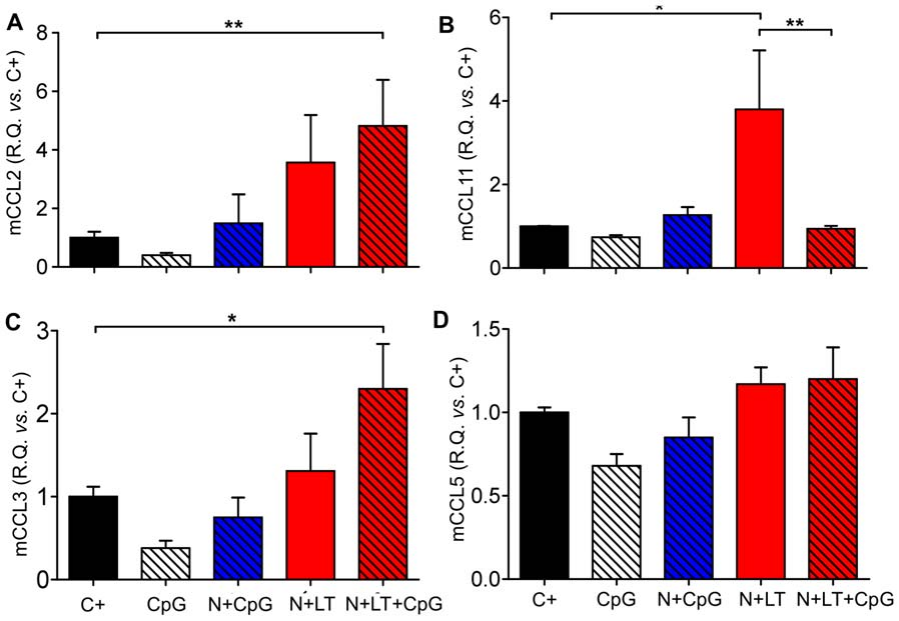
CpG added to the neonatal vaccine decreased pulmonary CCL11 mRNA expression upon RSV challenge. The level of expression of genes encoding mCCL2 (A), mCCL3 (C), mCCL5 (D) and mCCL11 (B) was monitored by quantitative real time PCR of RNAs extracted from individual lungs collected at d5 p.i. Data were normalized to the mHPRT and expressed relative to the unvaccinated infected control group (C+) (R.Q. = 100×2^−ΔΔCt^). Data are mean±SEM from n≥4 mice per group (Mann Whitney test, * p<0.05). Two independent experiments were done.

We then investigated whether vaccine formulation could modify the pattern of expression of several chemokines that could affect the Th1/Th2 balance and the recruitment of eosinophils. The levels of expression of mRNAs encoding the murine chemokines mCCL2 (MCP1), mCCL11 (Eotaxin-1), mCCL3 (MIP1α and mCCL5 (RANTES) were assessed by quantitative real time PCR in lungs collected 5 days after the viral challenge (Fig. 6). As opposed to mice administered CpG alone in the neonatal period, which tended to have reduced levels of all four chemokine transcripts after challenge, N+LT+CpG vaccinated mice had significantly increased levels of CCL2 and CCL3 mRNA (Fig. 6A and 6C), whereas N+LT vaccinated mice had significantly increased levels of CCL11 (Fig. 6B) compared to the other groups (N+LT *versus* C+ p<0.05; N+LT *versus* N+LT+CpG p<0.01). This observation is consistent with the recruitment of BAL eosinophils following challenge in N+LT vaccinated mice as opposed to their absence when CpG were added to the vaccine formulation. The level of expression of CCL5 was not significantly affected by vaccination (Fig. 6D).

**Figure 7:**
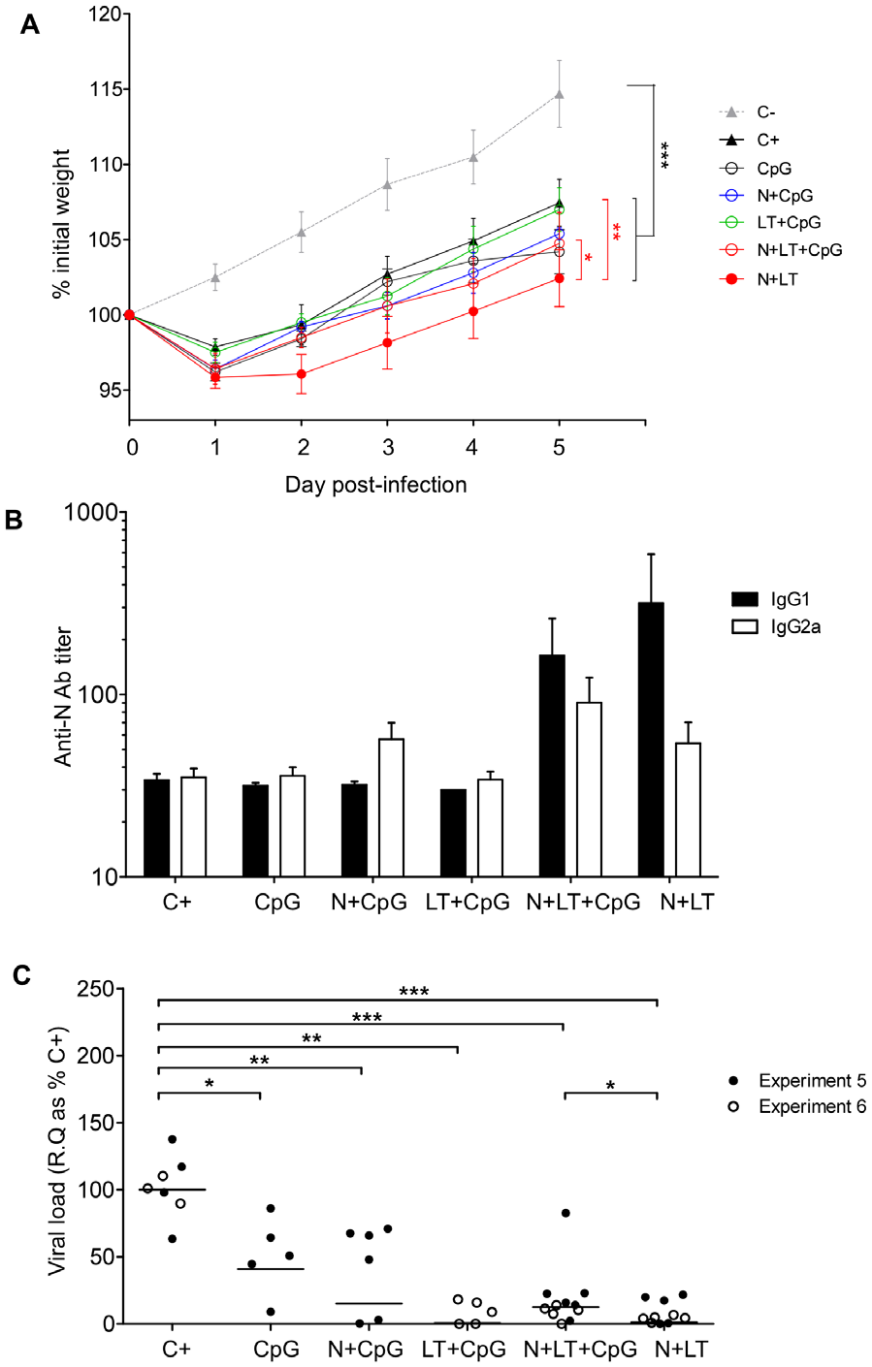
CpG added to the neonatal vaccine reduced weight loss, increased secondary IgG2a response and maintained viral protection upon RSV challenge. Mice vaccinated as neonates as indicated or non vaccinated littermates (C+) were sacrificed 5 days after the hRSV-A2 challenge. Data from 2 independent experiments combining different treatment groups are shown with ≥5 mice per group (C+: n = 7, CpG: n = 5, N+CpG: n = 6, LT+CpG: n = 5, N+LT: n = 10, N+LT+CpG: n = 11). (A) Mice were weighed daily from d0 till d5 p.i. and individual weight loss/gain was calculated as % of initial weight. Statistical analysis was performed to compare growth over the period d2 to d5 p.i. using the Tukey's multiple comparison test, repeated measures one way ANOVA (* p<0.05; ** p<0.01 and *** p<0.001). (B) IgG1 and IgG2a anti-N antibodies were titrated by ELISA in sera collected 5 days after the challenge. Data are mean±SEM from n≥5 mice per group. (C) Individual viral load in lungs: R.Q. of N transcripts, normalized to HPRT, are expressed as % of the mean of unvaccinated infected control group (C+) (R.Q. = 100×2^−ΔΔCt^). Data from 2 independent experiments combining different treatment groups are shown. Mann-Whitney U-test was used for comparison between treatments (* p<0.05; ** p<0.01; *** p<0.001 and **** p<0.0001).

We next tested whether addition of CpG to the vaccine formulation influenced weight loss, antibody production or viral load after an RSV challenge. Adding CpG to the neonatal N+LT vaccine abrogated the increase in weight loss after the RSV challenge (N+LT *versus* N+LT+CpG p<0.05) (Fig. 7A). N+LT primed mice produced mostly IgG1 anti-N antibodies as early as 5 days after the viral challenge (Fig. 7B). Addition of CpG to N or N+LT primed for an early rise in IgG2a anti-N antibodies, resulting in a more balanced IgG2a/IgG1 response in some mice, although the differences between groups were not statistically significant, due to highly variable individual titers.

Finally, as illustrated on figure 7C, neonatal administration of CpG alone significantly decreased the viral load in the lungs on day 5 p.i. (CpG *versus* C+ p<0.05) and combining CpG with LT even led to an almost complete “non-specific” protection against challenge (LT+CpG *versus* C+ p<0.01). Under these conditions, adding N to CpG or LT+CpG did not significantly improve protection although the level of protection elicited by N+LT vaccination remained slightly better (N+LT *versus* N+LT+CpG p<0.05) (Fig. 7C).

Thus efficient protection against RSV during the neonatal period could be achieved by combining the non-specific stimulatory effects of LT and CpG and antigenic priming with N.

## Discussion

In the present study we investigated in BALB/c neonate mice the safety and efficacy of a new mucosal vaccine candidate against RSV, which we previously validated in adult BALB/c mice [24]. BALB/c neonatal mice (≤7 days of age) have been considered as best approximating immune maturity of ≤2 months old babies [29] an age when RSV vaccination should be undertaken, since the most severe RSV disease affects mostly 2 to 6 month-old infants. Several studies addressing the issues of optimizing conditions for neonatal vaccination and, in peculiar, reducing the Th2 bias of neonatal immune responses have been performed using BALB/c neonates [16], [30]. Moreover BALB/c neonates constitutes a most sensitive experimental model for assessing immunopathological imprinting as generated by FI-RSV vaccination [28] or by an early RSV infection [26]: the propensity of the host to develop a skewed Th2 response to RSV at an early age set the stage for subsequent development of an asthma-like phenotype on re-exposure to the same virus [27], [31], [32].

Our present data show that neonatal mucosal vaccination with N+LT can generate efficient viral protection, resulting in over 95% reduction of the viral load, 5 days after a viral challenge with RSV at early adult age. Yet, this neonatal vaccination induced a prolonged state of hyper-reactivity to the virus, as shown by an increased weight-loss and an infiltration of the broncho-alveolar compartment by neutrophils and eosinophils a few days after the viral challenge. No such signs of vaccine-enhanced disease were observed in our first evaluation of the N+LT vaccine in adult BALB/c mice [24], which underscores the usefulness of the neonatal BALB/c mouse model for assessing the safety of a new RSV vaccine candidate, as previously stated by Plotnicky et al [28].

Individual weight-losses post-challenge were moderate (rarely over-passing 5–10% for 2–3 days), as compared to 15–20% weight-loss after challenge if mice had been primary infected as 4–6 days-old pups ([27] and our own unpublished data) or vaccinated as adults with M2 peptide with another mutant of LT (LT-K63) as adjuvant [33]. Yet, average weight-loss was significantly higher (over the period d2 to d5 p.i.) in the N+LT vaccinated group as compared to N-vaccinated or unvaccinated mice. As this suggested some vaccine (or adjuvant) exacerbated disease, we monitored lung inflammation by differential enumeration of the BAL leukocytes: clearly both the LT(R192G) adjuvant and, concerning eosinophils, the N^SRS^ antigenic component of the neonatal vaccine contributed to the recruitment of granulocytes in the broncho-alveolar space after the viral challenge, which did not occur in unvaccinated age-matched infected controls. Again, the magnitude of this inflammatory reaction was far below that seen in neonatally infected mice reinfected at adult age (e.g. eosinophilia reaching over 35% of BAL cells, [27], [31] and our own unpublished data). Comparing a large set of data from individual mice belonging to our different vaccination groups, we found a highly significant inverse correlation between individual viral load and % PMN cells in BAL (p<0.0001 for neutrophils, p<0.01 for eosinophils). This suggested that vaccination has set the stage for inflammatory responses that could contribute to protection upon viral challenge. Interestingly eosinophils have been proposed to promote RSV clearance [34] and were exonerated from contributing to vaccine-enhanced disease in experiments undertaken in adult mice [35], [36].

Anti-N antibodies were rarely detected in the serum of N or N+LT vaccinated mice one month after neonatal vaccination, but an early anti-N antibody response could clearly be induced in most N+LT vaccinated mice by an RSV challenge or by a boost with N. Thus, provided the LT adjuvant was co-administered, N^SRS^ was immunogenic in neonates and this N-specific antibody response could be boosted by an RSV infection. The almost exclusive production of IgG1 antibodies suggested that the immune memory resulting from neonatal N+LT vaccination was Th2 biased. Yet, we did not detect anti-N IgE antibodies upon neonatal vaccination, nor after the vaccine boost or viral challenge (data not shown), as was the case after a primary neonatal RSV infection followed by a challenge at adult age ([37] and our own unpublished data).

That neonatal i.n. vaccination with N+LT also primed for N-specific memory T cells was confirmed by the fact that spleen and respiratory lymph node cells isolated a week after a vaccine boost secreted both IFNγ and IL-5 upon *in vitro* restimulation with N. Once again the relatively high amount of the Th2 cytokine IL-5 secreted by local lymph node cells, suggested that the neonatal priming with N+LT had opened the way to a Th2 biased local memory.

Our experimental settings are very similar to those reported by VanCott *et al.* who vaccinated i.n. 7 d old BALB/c pups with the recombinant VP6 protein of EDIM rotavirus together with the same LT(R192G) adjuvant and induced a substantial level of protection against an oral EDIM virus challenge administered one month after vaccination [38]. Neither serum IgG/IgA nor stool IgA anti-VP6 antibodies could be detected in most mice before challenge, as was the case for anti-N antibodies in our experiments. However, splenocytes collected one month after the neonatal vaccination, before any vaccine boost or viral challenge, were shown to contain and/or secrete mostly IFNγ, and almost no IL-5, leading the authors to suggest that protection involved immune Th1 cells.

In many other studies, however, vaccination of BALB/c neonates with peptide or protein antigens led to Th2 biased immune responses [39]. Furthermore, primo-infection with hRSV in BALB/c neonates has also been shown to induce Th2 biased immune responses: mediastinal lymph node cells from mice primo-infected as pups respond with high IL-5 and reduced IFNγ secretion upon restimulation in culture with RSV infected, irradiated syngeneic splenocytes, in opposition to the responses of spleen or LN cells from mice primo-infected as adult [40].

Several mechanisms have been proposed at the molecular level to explain the Th2 bias of BALB/c neonates. First, the Th2 cytokine locus is hypomethylated during the neonatal period, thus allowing rapid high-level Th2 gene transcription (*IL-4*, *IL-5* and *IL-13*) [41]. Second, neonatal memory Th1 cells express high level of interleukin 13 receptor α1 (IL-13Rα1) which heterodimerizes with IL-4Rα, making Th1 cells prone to apoptosis driven by IL-4 produced by activated Th2 cells [42]. Finally, we have recently demonstrated that lung neonatal T cells have Th2 features and that the number of conventional and plasmacytoid dendritic cells is severely reduced in neonatal lungs [43].

Nevertheless it is possible to achieve Th1 immune responses in the neonatal context. For instance, several studies showed that addition of CpG to a neonatal vaccine can help to reduce the Th2 bias of the immune response [16], [17], [44]. Indeed adding CpG to our N+LT neonatal vaccine not only reduced the Th2 bias of the recall response to a boost with N (less IL-5, more IgG2a), but also abrogated eosinophilia following an RSV challenge, as assessed in BAL cells. These observations are highly consistent with the significant decrease in mCCL11 (eotaxin) mRNA that we have recorded in the lungs when CpG were added to the N+LT vaccine.

However addition of CpG to the N+LT vaccine did neither reduce the neutrophilia in BAL post-challenge, nor the inflammatory leukocytic infiltrates in lung sections. Correlations have been reported between the pattern of chemokine production and the severity of RSV disease in infants [45], [46] or with the Th1/Th2 immunopathological imprinting induced by RSV infection after prior sensitization to individual RSV proteins (F or G) in adult BALB/c mice [47]. According to these studies, the enhanced level of expression of CCL2 and CCL3 mRNA upon challenge of our N+LT and N+LT+CpG vaccinated mice, is consistent with mixed Th2 (increase in CCL2 mRNA) and Th1 (increase in CCL3 mRNA) inflammatory settings, and could explain the increased number of BAL neutrophils. Interestingly, CCL3 was recently shown to protect against TNFα-mediated weight-loss and illness after a primary RSV infection in adult mice [48].

Both N+LT and N+LT+CpG neonatal vaccination conferred highly significant reduction of the viral load. However, the level of protection achieved in the presence of CpG could be partly related to some innate effect on the neonatal immune system since CpG and even more so LT+CpG alone very significantly reduced lung viral load, extending a previously described observation by Cho and al. [49]. Nevertheless, N+CpG vaccinated mice were not protected against RSV replication as efficiently as were the N+LT or N+LT+CpG vaccinated mice, suggesting that a combination of the stimulation of innate immunity by CpG, favoring Th1 responses, and the stimulation of N-specific immunity by the LT adjuvant, both contributed to protection.

N^SRS^ are nanostructures which could by itself confer them innate immunostimulatory properties: indeed, protein nanocages of quite similar structure and size delivered intranasally to adult mice were shown to induce iBALT development and to confer protection against several respiratory viruses [50]. Accordingly, in our own experiments, histological examination of lungs collected early after the RSV challenge showed clear development of iBALT along the main bronchi, if mice had been vaccinated as pups with N+LT or with N+LT+CpG. Interestingly, neonatal mice were recently shown to be particularly prone to iBALT induction upon inflammatory (LPS or CpG) stimuli, with IL-17 being essential to iBALT development in the neonatal period [51].

Thus, although intranasal vaccination may seem a logical route for protection against respiratory pathogens because it will efficiently prime local immune effectors, it can also potentiate inflammatory responses through local priming of Th17 memory cells [52]. We did not look for these immune mediators in the present study but we are keen to evaluate other delivery route like the intradermal or transcutaneous routes, able to generate systemic and local immunity with increased safety and feasibility in the neonatal context [53]. Significantly, a double LT mutant (dmLT) with even lower toxicity than LT(R192G) has been developed [54]. Finally, we currently explore whether our N^SRS^ nanoparticles could be used as carrier to improve immunogenicity of peptides encoding major RSV neutralizing epitopes from F and G envelope proteins.

Thus we believe that a safe and efficient pediatric vaccine against RSV will require innovative solutions through adapted combination of antigen, adjuvant and delivery route. Our present work confirms that safety and efficacy of RSV vaccine candidates should be evaluated in the most sensitive neonatal models.

## Materials and Methods

### Ethics statement

All animal experiments were carried out under the authority of licence issued by the Direction des Services Vétérinaires (accreditation number 78–115 to A. P-C.) and approved by the ethics committee COMETHEA (COMité d'ETHique appliqué à l'Expérimentation Animale INRA Jouy-en-Josas et AgroParisTech): authorization number 11/036. All efforts were made to minimize suffering.

### Vaccine formulation and virus

The production and purification of the recombinant N protein from hRSV (Long strain) in *E. coli*, as sub-nucleocapsid ring structures of 10–11 monomers of N bound to random stretches (70 bases) of bacterial RNA (N^SRS^ refered as N) have been described previously [23], [24]. For use as a vaccine, an additional filtration on EndoTrap red® columns (Hyglos GMBH, Germany) reduced endotoxin contamination to <1 EU/mg protein (QCL-1000 LAL assay, Cambrex).

The detoxified mutant *E. coli* enterotoxin LT(R192G), provided endotoxin free by Prof. Clements (Tulane University, New Orleans, USA) was kept sterile at 4°C. Phosphorothioate- CpG oligodeoxynucleotides (ODN) 1826 (TCCATGACGTTCCTGACGTT) was purchased from Sigma Genosys, resuspended in injectable water as a 1 mM stock solution and kept frozen at −20°C.

The hRSV strain A2 (provided by Prof. Openshaw, Imperial College, Saint Mary's Hospital, London) was propagated and titrated by plaque assay on HEp-2 monolayers using avicel-containing overlay media to control virus spread. Five days after infection, lived cells were stained with crystal violet and plaque forming units (pfu) were numerated.

### Animals and immunization-challenge protocols

Male and female BALB/c mice purchased from the Centre d'Elevage Janvier (Le Genest, St Isle, France) were bred and housed under FELASA pathogen-free conditions in our animal facilities (IERP, INRA, Jouy-en-Josas).

Five to seven days old pups of both sexes were vaccinated by intranasal instillation of 8 to 10 µL of 0.9% endotoxin-free NaCl, containing or not 3 µg N^SRS^ and/or 2 µg LT(R192G) and/or 2 nmoles (12.7 µg) CpG ODN 1826 (Sigma-Aldrich). Each treatment was administered to 4 to 8 pups from at least 2 different litters, in order to minimize individual variations.

Since a limited number of pups could be included, handled and infected at the same time in any given experiment, all desired control groups could not be included in each experiment. Hence, experimental data presented in the figures usually comprise data from two or more independent experiments.

At 5 to 6 weeks of age, mice were anesthetized by an intraperitoneal injection of Avertine (2.5 mg/g of body-weight of 2-2-2-tribromoethanol, Aldrich) and received, by intranasal instillation either a vaccine boost (10 µg N^SRS^ in 25 µL of saline), or a challenge RSV infection (50 µL, 5.10^6^ p.f.u. of hRSV-A2). Individual daily weight was recorded until mice were killed 5 or 8 days after challenge, which allowed us to monitor the viral load, the weight loss and the onset of the antibody response and lung inflammation in the same individual mice.

### Sample collection

Sera were obtained from blood collected via retro-orbital puncture before (d0) and 7 days after a boost immunization, or 5 to 8 days after a challenge infection.

Mice were killed by cervical dislocation and the spleen and local lymph nodes (LN) (cervical and maxillary LN draining the upper respiratory tract and mediastinal LN) were dissected out in complete RPMI medium (RPMI 1640, Gibco BRL, supplemented with 10% heat-inactivated FCS, 2 µM L-Glutamine, Penicillin and Streptomycine).

The left bronchus was clamped and the left lobe of the lungs was snap-frozen in liquid nitrogen, and kept frozen at −80°C until processed for RNA extraction for qRT-PCR assays.

Broncho-alveolar lavages (BAL) were performed by flushing the other lobes of the lungs via tracheal puncture four times in and out with 0.7 mL Ca^2+^ and Mg^2+^-free PBS supplemented with 1 mM EDTA. BAL fluids were centrifuged and supernatants (BALF) were stored frozen at −20°C for ELISA assays of anti-N antibodies or cytokines. BAL cells were resuspended in complete RPMI medium and adjusted to appropriate concentrations for cytocentrifugation or FACS analysis.

Alternatively, the lungs were gently filled with 0.5 mL of 10% paraformaldehyde (PFA) via the trachea and preserved in 10% PFA at 4°C until processed for histological examination.

### Determination of pulmonary RSV viral load and chemokine expression by quantitative Real-Time PCR

Frozen lungs (left lobes) were resuspended in 0.6 mL lysis buffer (Qiagen RTL buffer plus 0.1% 2-mercaptoethanol, Merck) in V bottomed microtubes containing 0.1 mL of 1 to 1.2 mm ceramic beads (Mineralex, Bron, France) (both RNAse free) and vortexed for 15 s at 6000 rpm using a bead grinder homogenizer (Precellys 24, Bertin Technologies, Ozyme, St Quentin en Yvelines, France). Total RNA were extracted from lung homogenate using RNA minikit columns (Qiagen), with a 15 min DNase treatment (RNase free DNase set, Qiagen), according to the manufacturer's instructions. Eluted RNA were quantified at 260 nm using a Nanodrop (Labtech, France). Individual RNA samples (4 µg) were reverse transcribed for 1 h at 42°C, using 300 U of M-MLV Reverse Transcriptase (SuperScript II, Invitrogene) with 7 µM of random hexanucleotide primers (pd(N)_6_, Pharmacia Biotech), 15 nmol of each dNTP, 5 mM DTT and 60 U of ribonuclease inhibitor (RNaseOUT, Invitrogen) according to the manufacturer's instructions.

The primers (from Sigma–Aldrich, aliquoted and stored frozen as 100 µM solutions at −20°C) used to target either a conserved region of the *N* gene (position 42 to 125 of huRSV A2, [24]), or the murine *HPRT* gene, or the *CCL2*, *CCL3*, *CCL5* and *CCL11* murine chemokine genes (design with http://mouseprimerdepot.nci.nih.gov/) are listed in Table 3.

**Table 3:** List of primers used for quantitative real time PCR.

Name of gene	Forward primer (5′ to 3′)	Reverse primer (5′ to 3′)
N (huRSV-A2)	AGATCAACTTCTGTCATCCAGCAA	TTCTGCACATCATAATTAGGAGTATCAAT
mHPRT	CAGGCCAGACTTTGTTGGAT	TTGCGCTCATCTTAGGCTTT
mCCL2	GGGATCATCTTGCTGGTGAA	AGGTCCCTGTCATGCTTCTG
mCCL3	GATGAATTGGCGTGGAATCT	CTGCCCTTGCTGTTCTTCTC
mCCL5	CCCACTTCTTCTCTGGGTTG	GTGCCCACGTCAAGGAGTAT
mCCL11	TAAAGCAGCAGGAAGTTGGG	CATCTGTCTCCCTCCACCAT

Real time PCR was run in 96 well microplates on a Perkin Elmer ABI Prism 7900HT Sequence Detector. For determination of the viral load or of *CCLx* gene expression, individual cDNA (1 µL diluted in 10 µL RNAse-free water) were mixed with 15 µL SYBR GREEN PCR Master Mix (Applied Biosystems) containing 300 nM of both N primers, 500 nM of both CCLx primers or 500 nM of both HPRT primers, in triplicate for each gene. Non-template controls were run in each assay. Fluorescence curves were analyzed using the software Sequence Detector System (SDS 2.3, Perkin-Elmer) to determine the cycle threshold (Ct) values for each gene. Individual data were normalized to HPRT, by calculating the ΔCt value {Ct(N or CCLx)median -Ct(mHPRT)median} and the ΔΔCt {sample ΔCt - mean ΔCt of at least 3 unvaccinated infected controls (C+)} were calculated for each sample. Viral load was expressed as % of unvaccinated infected controls (using the formula R.Q. = 100×2^−ΔΔCt^). The viral load data from our qRT-PCR assay showed strong correlation with those from our tissue culture plaque assay (Fig. S1, r = 0.99, p<0.001).

Similarly, the expression of the chemokine transcripts normalized to mHPRT transcripts are expressed relative to unvaccinated infected controls (C+) (R.Q. = 2^−ΔΔCt^).

### May-Grünwald-Giemsa staining and histology

BAL cells from individual mice, were numerated, spread on duplicate microscope slides (Superfrost, Thermo, France) by cytocentrifugation (5 min, 700 rpm, Cytospin 5, Shandon, France) and stained with May-Grünwald and Giemsa. At least 400 leukocytes were counted blindly for each sample.

Lungs collected d8 p.i. were fixed in 10% paraformaldehyde, embedded in paraffin and 5 µm sections, stained by hematoxylin, eosin and saffron were photographed using a Nanozoomer (Hamamatsu).

### N-specific antibody E.L.I.S.A

Individual mouse sera and BALF were assayed for N-specific antibodies (Ig(H+L), IgG2a, IgG1 or IgA) by ELISA as previously described in [24]. End-point antibody titers were calculated by regression analysis, plotting dilution versus A450 using Origin software (regression curve y = (b+cx)/(1+ax). Endpoint titers were defined as the highest dilution resulting in an absorbance value twice that of non-immune control sera or BALF.

### IFN-γ and IL-5 production by immune spleen or lymph node cells

Red-blood cell depleted leukocyte suspensions were prepared as previously described [24] from individual spleens or lymph nodes (LN: cervical and mediastinal pooled from 2 to 3 mice) collected 7 days after a boost with N. Spleen or LN cells resuspended in RPMI complete medium and 50 µM 2-Mercaptoethanol were seeded in 96 wells microplates (4.10^5^ cells/well) and co-cultured in triplicates with N (10 µg/mL) or PMA-Ionomycin (10 ng/mL and 1 µg/mL, respectively) or culture medium. After 72 h at 37°C with 5% CO_2_, supernatants for each condition were pooled and stored frozen at −20°C.

Supernatants were assayed for IFNγ and IL-5 by standardized ELISA assays as previously described [24]. Briefly, for IFNγ we used mAb R4-6A2 for capture and biotinylated XMG1.2 for revelation (BD biosciences) and murine rIFNγ (R&D systems) diluted from 3300 to 1.5 pg/mL in duplicate wells to establish a standard curve. For IL-5, we used mAb TRFK5 for capture and biotinylated TRFK4 (BD biosciences) for revelation and murine rIL5 (R&D systems) diluted from 1000 to 7.5 pg/mL in duplicate wells to establish a standard curve. Cytokine concentrations were determined using the Revelation software (Dynex).

### Flow cytometry

After a first 20 min blocking step using anti-CD32/CD16 (FcBlock, BD bioscience, 5 µg/ml or 10 µg/ml for highly inflammatory BAL collected post-infection), BAL cells were labeled with anti-CD45-PerCP, anti-Siglec-F-PE and anti-CD11c-biotin, followed by streptavidin-APC (all from BD Pharmingen).

Cells were then fixed in 10% Cellfix (BD bioscience) and data, acquired on at least 5,000 CD45+ leukocytes with a FACSCalibur (BD Biosciences), were analyzed with Flow Jo software. After gating on CD45^+^ leukocytes and excluding autofluorescent cells, BAL eosinophils were gated as Siglec-F**^+^**, CD11c^lo/−^ according to [55].

### Statistical data analysis

Data were expressed as arithmetic mean ± standard error of the mean (SEM). Non-parametric Mann-Whitney (two groups), ANOVA Tukeys multiple comparison test (>2 groups) or Spearman correlation statistical tests were used to compare unpaired values (GraphPadPrism software). Values of p<0.05 were considered significant, levels of significance are indicated on the graphs with stars : * p<0.05; ** p<0.01; *** p<0.001; and **** p<0.0001.

## Supporting Information

Figure S1 **Correlation between viral load titration by plaque assay and q-RT-PCR.** Five infected and two non-infected adult mice were sacrificed 4 days after the hRSV-A2 challenge. The lungs were cut in two equal parts and the viral load was titrated either by plaque assay on HEp-2 monolayers, or by qRT-PCR: R.Q. of N transcripts, normalized to HPRT, are expressed as % of the infected mice (C+) (R.Q. = 100×2^−ΔΔCt^). Non parametric Spearman correlation test was used to calculate a correlation coefficient (r), (*** p<0.001).
(TIF)
