# Supplementary figures and images for: Nucleoprotein Nanostructures Combined with Adjuvants Adapted to the Neonatal Immune Context: A Candidate Mucosal RSV Vaccine
Source: PLoS One. 2012 May 24;7(5):e37722. doi: 10.1371/journal.pone.0037722 (PMC3359995; doi:10.1371/journal.pone.0037722)

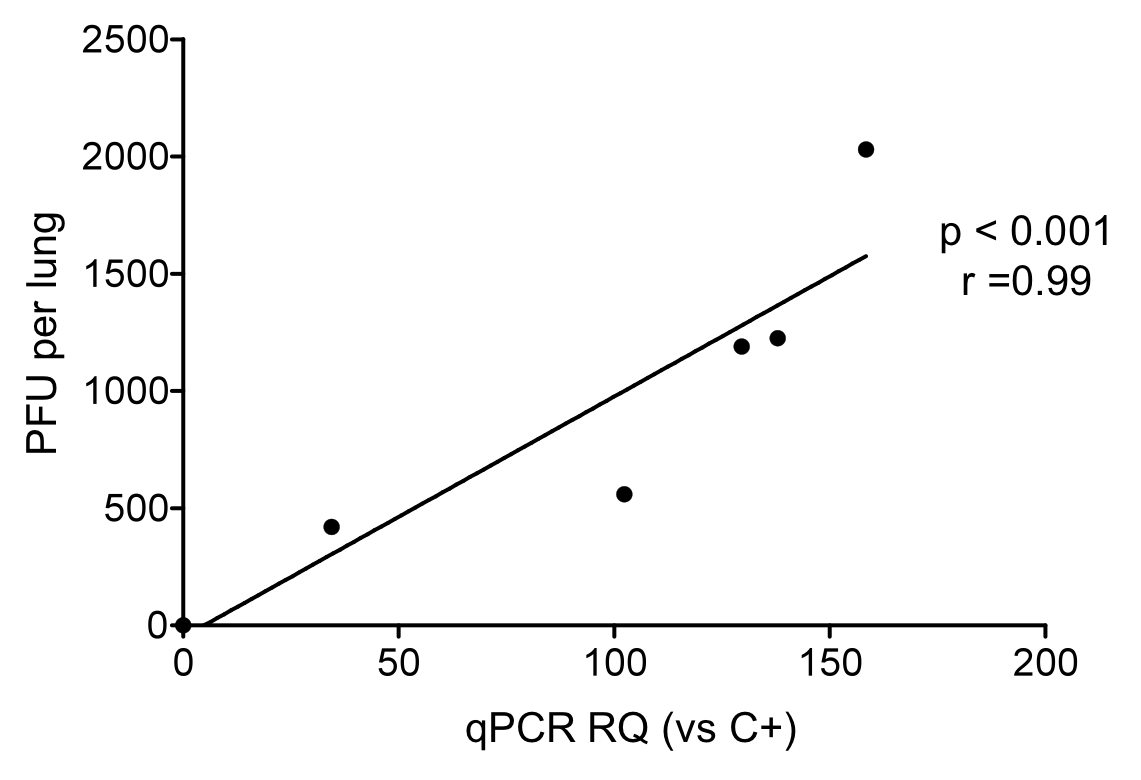

Supplement: Figure S1 — Correlation between viral load titration by plaque assay and q-RT-PCR. Five infected and two non-infected adult mice were sacrificed 4 days after the hRSV-A2 challenge. The lungs were cut in two equal parts and the viral load was titrated either by plaque assay on HEp-2 monolayers, or by qRT-PCR: R.Q. of N transcripts, normalized to HPRT, are expressed as % of the infected mice (C+) (R.Q. = 100×2−ΔΔCt). Non parametric Spearman correlation test was used to calculate a correlation coefficient (r), (*** p<0.001). (TIF) [file pone.0037722.s001.tif]
